# HPV16 Impacts NHERF2 Expression in Oropharyngeal Cancers

**DOI:** 10.3390/pathogens12081013

**Published:** 2023-08-03

**Authors:** Lucija Lulić, Antonia Jakovčević, Iva Kovačić, Luka Manojlović, Emil Dediol, Josipa Skelin, Vjekoslav Tomaić

**Affiliations:** 1 Division of Molecular Medicine, Ruđer Bošković Institute, Bijenička 54, 10000 Zagreb, Croatia; 2 Clinical Department of Pathology and Cytology, University Hospital Center Zagreb, 10000 Zagreb, Croatia; 3 Department of Pathology and Cytology, University Hospital Dubrava, 10000 Zagreb, Croatia; 4 Department of Maxillofacial Surgery, University Hospital Dubrava, 10000 Zagreb, Croatia

**Keywords:** HNSCC, OPSCC, HPV, E6, p16, NHERF2

## Abstract

Infection with human papillomaviruses (HPVs), in particular with HPV type 16, is now considered to be a key risk factor for the development of a subset of oropharyngeal squamous cell carcinomas (OPSCC) that show different epidemiological, clinical, and prognostic characteristics from HPV-negative (HPV−) OPSCCs. So far, extensive research efforts aiming to distinguish these two distinct entities have not identified specific biomarkers, nor led to different therapies. Previous research has shown that HPV16 E6 oncoprotein binds NHERF2, inducing its proteasomal degradation, and consequently increasing cell proliferation; we therefore aimed to investigate how this might be reflected in human histological samples. We analyzed NHERF2 expression patterns in HPV16-positive (HPV16+) and HPV− OPSCC samples, to investigate any potential differences in NHERF2 pattern. Interestingly, we observed a statistically significant decrease in NHERF2 levels in HPV16+ and poorly differentiated HPV− OPSCCs, compared with healthy tissue. Furthermore, we observed a significant reduction in the percentage of NHERF2 immunoreactive cancer cells in HPV16+ tumors, compared with well and moderately differentiated HPV− OPSCCs, suggesting the importance of 16E6’s targeting of NHERF2 in HPV-driven oncogenesis in the head and neck area.

## 1. Introduction

Head and neck squamous cell carcinomas (HNSCC) are the most prevalent type of head and neck cancers (HNC), accounting for over 90% of cases. They originate from the mucosal epithelium of the upper aerodigestive tract and comprise cancers of oral cavity, nasopharynx, oropharynx, larynx, and hypopharynx [1,2]. According to GLOBOCAN’s data from 2020, HNCs represented approximately 5% of all human cancer cases, with 878,348 new cases and 444,347 deaths reported. Long-term surveillance of HNSCC epidemiology has revealed an increase in both incidence and mortality rates [3]. Numerous risk factors have been identified through epidemiological research, including tobacco use, alcohol abuse, exposure to carcinogens, poor oral hygiene, family and/or medical history, diet, and infection agents [1,4,5]. While tobacco and alcohol are the primary contributors to HNSCCs, affecting the oral cavity, larynx, and hypopharynx, their impact on oropharyngeal tumor formation is relatively minor. Instead, the presence of oncogenic human papillomavirus (HPV), specifically type 16, has been recognized as a significant cause, accounting for up to 70% of oropharyngeal cancers [5]. HPV-positive (HPV+) and HPV-negative (HPV−) HNSCCs can be considered as two separate diseases due to the differences in various aspects, such as the location they originate from, the mechanisms of their development, and the factors that increase the risk of occurrence [6]. The vast majority of HPV+ HNSCCs occur in the oropharynx, especially in males of younger age, whereas HPV− ones arise more commonly in the oral cavity, larynx, or hypopharynx [1,7]. Genomic analyses have also revealed extremely high heterogeneity in HNSCC in terms of molecular signature. In HPV+ cancers p53 and pRb, tumor suppressors are inactivated due to interactions with viral oncoproteins E6 and E7 [8,9], respectively, while mutations in mentioned genome guardians are often found in HPV− HNSCCs [10,11]. Also, HPV+ cancers are more likely to be smaller and poorly differentiated with cystic lymph node metastasis, but despite a more aggressive clinical presentation, these patients have greater response to chemotherapy and radiation, possibly due to the presence of an intact p53 or better loco-regional control [6].

HPVs are small, non-enveloped DNA viruses strongly implicated in the development of various cancer types. To date, nearly 220 HPV genomes have been sequenced and sorted into five genera. The well-characterized *Alphapapillomavirus* (αHPV) species have been shown to preferentially infect anogenital and oral mucosal epithelia [12], and are classified as either high-risk (HR, e.g., HPV16, HPV18) or low-risk (LR, e.g., HPV6, HPV11), according to their capacity to cause malignant transformation [13]. HR HPVs are associated with almost 100% of cervical cancers, with the most oncogenic HPV16 and HPV18 being linked to about 70% [14,15]. In recent decades, cancers arising in the oropharynx, especially at the back of the tongue and in the tonsils, have also been associated with some HR HPVs. Unlike cervical cancers, oropharyngeal cancers are primarily attributable to HPV16, with much less association with HPV18 and rarely with other HR types [10,16]. Although HPV infections are the most common sexually transmitted infections, the immune system usually recognizes the afflicted cells and neutralizes them in less than two years from the initial infection, and this is defined as an absence of HPV DNA detection on serial follow-up swabs [17]. However, when the infection persists for a longer time, under circumstances that are still not clear, viral DNA is randomly integrated into the chromosomes of affected cells, resulting in the disturbance of E2, ultimately causing the elevation of E6 and E7 expression [18,19]. In the case of HNSCCs, on the other hand, E2 is typically hindered through DNA methylation, leading to a persistent and unregulated expression of E6 and E7 [11,20,21]. This process, from primary infection to the development of cancer, is well defined in the cervix. Two main viral oncoproteins, E6 and E7, are initiators of HPV-driven malignancies. They cooperatively target different cellular pathways involved in cell cycle regulation and apoptosis [22,23], primarily by binding, and targeting for proteasome-mediated degradation, the tumor suppressors p53 and pRb, respectively [8,24,25,26]. In addition to promoting p53 degradation, numerous studies have shown that cancer-causing E6 targets many other cellular substrates, including proteins that contain PDZ (PSD-95/Dlg/ZO-1) domains. These domains are protein interaction modules involved in maintaining cellular homeostasis [27,28]. Many of them, including DLG1, DLG4, SCRIB, MAGI, and PTPN13, in addition to being tumor suppressors, are also responsible for the regulation of cell polarity and cell–cell contacts [27,29]. Interestingly, a cellular PDZ domain-containing protein named Na+/H+ Exchanger Regulatory Factor 1 (NHERF1) was reported as a degradation target for E6 proteins from both HR and LR HPVs [30,31]. As recently described, alterations in NHERF1 protein levels were observed in HPV16+ pre-malignant and malignant anogenital lesions, but not in lesions induced by other HR HPV types or by LR types [32]. The related, and structurally similar, protein NHERF2 contains two PDZ domains which enable it to simultaneously bind several cytoplasmic and membrane proteins, to organize ion transporters and transmembrane receptors on the apical membrane of epithelial cells, and to participate in various cellular processes [33]. NHERF2 is also expressed in endothelial cells, where it acts as a key negative regulator of proliferation; cells with downregulated NHERF2 have increased proliferation and accelerated cell cycle, even in the absence of growth factors [34]. The ablation of NHERF2 protein expression appears to have a number of effects, including increased calcium concentration in the cytoplasm and increased expression of c-Myc and cyclin D1, but also decreased expression of CDK (cyclin-dependent kinase) inhibitor p27, which is characteristic of many cancers [35,36]. Additionally, NHERF2 was recently shown to be a cellular substrate of HR E6 oncoproteins, which target NHERF2 via their C-terminal PDZ-binding motif (PBM). This was first shown in overexpression assays HEK293 cells, with both HPV16 and 18 E6, and then further confirmed in HPV18+ HeLa cells, and HPV16+ CaSki and SiHa cells. As reported, E6 oncoproteins from HPV16 and HPV18 target NHERF2 for a proteasome-mediated degradation, leading to p27 downregulation and, consequently, to increased cellular proliferation [37].

Although the last two decades have yielded numerous studies that have significantly increased our general knowledge concerning the role of HPV in HNSCCs, insights for the generation of novel strategies for their prevention and early diagnosis are still missing. So far immunohistochemical (IHC) identification of p16 tumor suppressor is used as a substitute marker for HPV+ cancers since E7 oncoprotein stimulates its overexpression [38]. However, no strict correlation between p16 overexpression, HPV infection, and the progression of cancer has been established. Studies have shown that about 10–20% of OPSCCs that are p16-positive are not HPV infected [39,40]. Thus, we aimed to better understand the process of HPV-driven oncogenesis in the head and neck area by investigating potential changes in the expression patterns and localization of NHERF2 in the presence of HPV16. Such knowledge could be important for distinguishing HPV− from HPV16+ OPSCCs in the early stages of disease development. 

## 2. Materials and Methods

### 2.1. Tissue Samples

The study was conducted on FFPE samples (*n* = 48), grouped as either HPV16+ oropharyngeal cancer (*n* = 19), HPV− oropharyngeal cancer (*n* = 29), or healthy uninfected tonsillar tissue (*n* = 8). The tissue samples were obtained from the archives of the Department of Pathology and Cytology, University Hospital Dubrava (ethical permit no. BEP-55 48/2-2016) and the Department of Pathology and Cytology University Hospital Centre, Zagreb, Croatia (ethical permit no. 02/21 AG, Class 8.1-21/15-2). All the tissue samples were previously fixed in 10% buffered formalin and embedded in paraffin. 

### 2.2. DNA Isolation and Genotyping

From each FFPE sample, five to seven slices (10 µm) were used for DNA isolation, utilizing a commercial kit (NucleoSpin^®^ DNA FFPE XS, Macherey–Nagel, Dueren, Germany) according to the manufacturer’s instructions. The concentration of the isolated DNA was measured using NanoPhotometer^®^ N60 (Implen GmbH, Munich, Germany), and the quality was validated by PCR using primers generating 99 bp long beta-actin fragments [40,41].

For HPV DNA detection, PCR was performed using short primers suitable for FFPE tissue samples GP5/6 and SPF 10, generating approximately 142 bp and 65 bp long PCR products, respectively [42,43]. PCR amplification was performed as previously described [40,44] and 10 μL of amplified PCR products were run on 3% agarose gels (Sigma Aldrich, St. Louis, MO, USA). A sample was considered to be HPV+ if either the GP5/6 or SPF10 PCR was positive. Additionally, HPV16+ samples were distinguished using a supplementary primer pair generating a shorter DNA sequence of 98 bp, as previously described [45].

### 2.3. Immunohistochemistry

Sections (7 µm) of the FFPE tissue sections were mounted on pretreated glass slides, deparaffinized in the xylene substitute BioClear (Biognost, Zagreb, Croatia), and rehydrated using a graded ethanol series of 100%, 95% and 70%), respectively. The p16 expression was analyzed using the CINtec^®^ Histology kit system (Roche Holding AG, Basel, Switzerland) according to the manufacturer’s instructions, and was compared with the HPV status of the corresponding tissue. Endogenous expression of NHERF2 was detected using EnVision^®^ + Dual Link System-HRP (Agilent, Santa Clara, CA, USA) according to the manufacturer’s instructions, and mouse monoclonal anti-NHERF2 antibody (C-2, Santa Cruz Biotechnology, Inc., Dallas, TX, USA) at 1:30 dilution. As a negative control, primary antibody was replaced with the provided negative control solution (CINtec^®^ Histology kit system) and antibody diluent (10% FBS, 1% BSA, 0.3% Triton-X TBS) for p16 and NHERF2 staining, respectively. The intensity of NHERF2 immunostaining was graded and scored by a pathologist as following: 0 (no staining), 1+ (low intensity), 2+ (medium intensity), and 3+ (strong intensity).

### 2.4. Statistical Analysis

Descriptive statistics were used to evaluate differences in NHERF2 expression and localization in healthy uninfected tonsillar tissue, HPV16+, and HPV− OPSCCs. The Kruskal–Wallis test was used to evaluate possible significant differences between the designated groups, HPV− OPSCCs grades 1–3 and HPV16+ OPSCCs with Dunn’s multiple comparison post-test. The ratio of NHERF2-positive staining in cancer cells was an estimation set by a pathologist. All statistical analyses were performed using GraphPad Prism 8 software.

## 3. Results

### 3.1. The Expression of p16

In this study we examined 48 formalin-fixed paraffin-embedded (FFPE) OPSCC tissue samples, and 8 healthy uninfected tonsillar tissue samples were used as controls. We isolated DNA and performed HPV genotyping by PCR as previously described [40]. The results showed that there were 19 HPV16+ and 29 HPV− OPSCC samples, which were then further graded by a pathologist as either G1, or well differentiated (7/29); G2, or moderately differentiated (8/29); and G3, or poorly differentiated to undifferentiated (14/29). 

**Figure 1:**
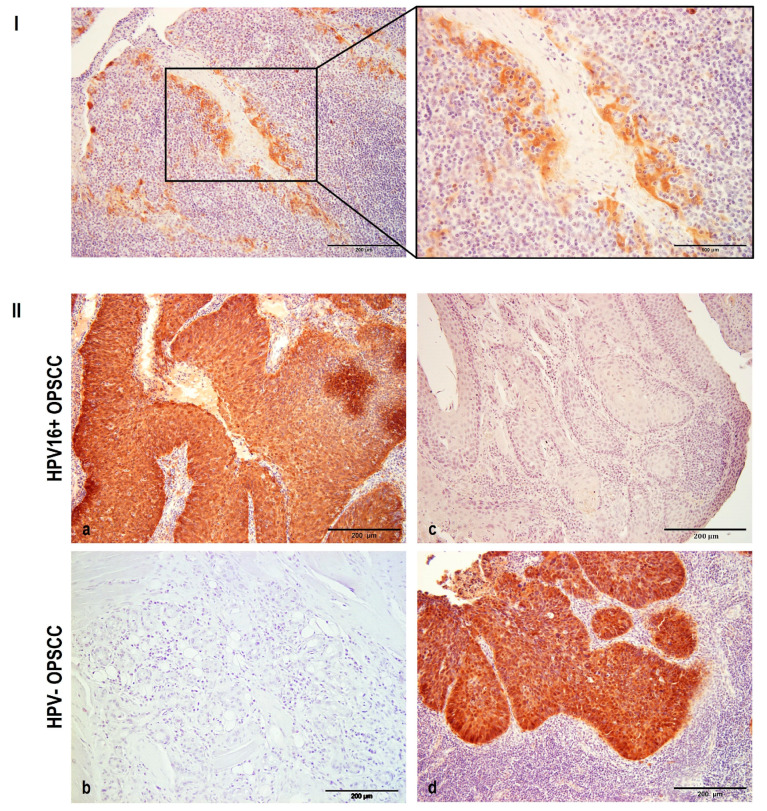
Immunohistochemical analysis of p16 expression. FFPE samples were immunostained for p16, counterstained with hematoxylin and visualized by light microscopy. Representative images: (**I**) Positive p16 immunoreactivity was observed solely in the reticular crypt epithelium of healthy uninfected tonsils. (**II**) (**a**) The majority of HPV16+ OPSCC samples showed p16+ immunoreactivity. (**b**) A small proportion of HPV16+ cancers stained as p16−. (**c**) No p16 immunoreactivity was detected in the majority of HPV− OPSCCs. (**d**) p16 immunoreactivity was observed in a few HPV− OPSCCs. Scale bars = 200 μm and 100 μm (**I**); 200 μm ((**II**) (**a**–**d**)).

To investigate and link p16 protein expression with tumor grades and/or HPV status, p16 IHC staining was performed in all samples (Figure 1). Positive p16 (p16+) immunoreactivity was detected in healthy uninfected tonsils exclusively in the reticular crypt epithelia, but not in the surrounding tissue (Figure 1I), which is in line with previously published data [46]. As expected, p16+ immunoreactivity was observed in almost 90% of HPV16+ OPSCC samples. However, p16+ staining was not HPV16 exclusive, since approximately 10% of HPV– OPSCC samples also showed p16 positivity (Figure 1II). This is in accordance with studies published so far, in which p16 staining showed about 10–15% discrepancies, regardless of HPV presence [39,47]. 

### 3.2. NHERF2 Expression Intensity and Score Are Dependent on Tumor Grade and HPV16 Positivity

**Figure 2:**
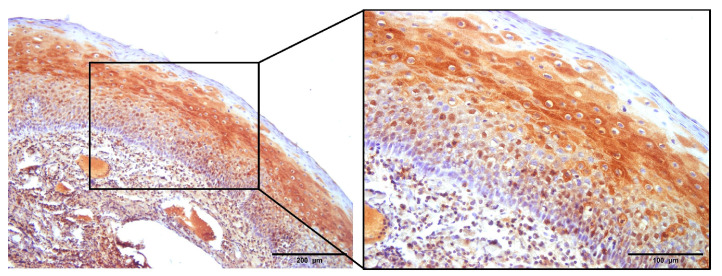
NHERF2 expression and localization patterns in control uninfected tonsils. FFPE samples were immunostained with anti-NHERF2 antibody, counterstained with hematoxylin and visualized by light microscopy. In uninfected control tonsillar tissue, positive NHERF2 immunoreactivity was detected mostly in the cytoplasm of the cells in the middle layer of the stratified squamous epithelium. Scale bars = 200 μm and 100 μm.

Prior to studying NHERF2 expression patterns in cancer samples, its expression and localization were investigated in healthy, uninfected tonsillar tissue. NHERF2 was detected in all eight FFPE samples, with slight differences in immunostaining patterns. It was mostly detected in the stratified squamous epithelium of the tonsils, mainly in the middle layer of the stratified epithelium and occasionally in the basal layers, but it was almost always absent from the surface layer of the differentiated epithelium (Figure 2). In all the control samples, NHERF2 was mostly localized in the cytoplasm of epithelial cells, and in two samples its presence was also observed in cell membranes. Predominantly strong or medium staining intensity was detected, with a weak intensity in only one sample. 

**Figure 3:**
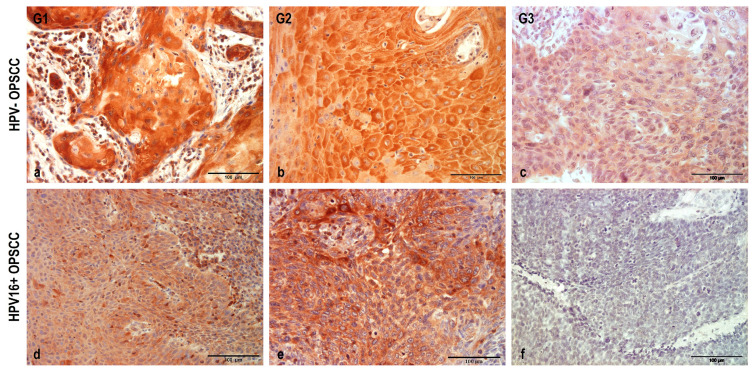
NHERF2 expression and localization patterns in HPV− and HPV16+ OPSCCs. FFPE samples were immunostained with anti-NHERF2 antibody, counterstained with hematoxylin, and visualized by light microscopy. Representative images: (**a**) High intensity cytoplasmic staining of NHERF2 in G1 HPV− OPSCCs; (**b**) Medium intensity cytoplasmic staining of NHERF2 in G2 HPV− OPSCCs; (**c**) Low intensity cytoplasmic staining of NHERF2 in G2 HPV− OPSCCs; (**d**) Low intensity cytoplasmic NHERF2 immunoreactivity in the majority of HPV16+ samples; (**e**) Moderate -intensity cytoplasmic NHERF2 immunoreactivity in a proportion of HPV16+ samples; (**f**) Lack of NHERF2 immunoreactivity in HPV16+ samples. Scale bars = 100 μm.

The NHERF2 expression profile was then investigated in HPV− OPSCCs, where it was detected in all analyzed samples, but one. Representative samples of all tumor grades are shown in Figure 3. In G1 OPSCC (Figure 3a), NHERF2 was detected in all samples predominantly as high-intensity staining (4/7 samples), similar to that observed in the control samples, and it was mostly accompanied by a large proportion of immunoreactive cancer cells. Medium staining intensity was observed in 3/7 samples, typically accompanied by a smaller proportion of immunoreactive tumor cells. In all samples, NHERF2 was localized in the cytoplasm with a negligible number of positively stained cell membranes observed. In the G2 tissue, staining patterns similar to those in G1 were observed. Exclusively cytoplasmic staining of mostly medium intensity was noted (Figure 3b), while 2/8 samples exhibited strong intensity similar to that in the control tissue and 1/8 samples presented weak intensity. The immunoreactivity of cancer cells was high on average, similar to that in G1 samples. Among the G3 samples, intermediate staining intensity (9/14) predominated (Figure 3c), with a few samples demonstrating weak intensity (4/14) and one sample in which no NHERF2 protein was detected at all. None of the G3 samples exhibited a strong staining intensity, comparable to that of the control samples. This was accompanied by a smaller proportion of immunoreactive cancer cells, with large deviations observed, from a very high (95%) to a very low (20%) percentage of staining. There was no change in the protein localization compared with the lower graded tumors, and NHERF2 was detected solely in the cytoplasm. In short, a gradual decrease in NHERF2 levels accompanied the decrease in cellular differentiation of HPV− cancers, whilst the protein localization remained the same between different tumor grades.

Since NHERF2 was characterized as a cellular target of HPV16 E6 [37], we were also interested in investigating its expression and localization in HPV16+ OPSCC, (Figure 3d–f) in the same manner as in HPV− OPSCC and healthy controls. Positive NHERF2 immunoreactivity was observed in almost all samples, with the majority of them showing lower-intensity staining (Figure 3d), while the remaining exhibited medium-intensity staining (Figure 3e). The intensity was noticeably weaker than in healthy tonsillar tissue and, on average, it was weaker than in G3 HPV− OPSCC. In only one sample, no NHERF2 immunoreactivity was seen (Figure 3f), which was possibly the result of complete degradation of the protein. The percentage of immunoreactive cancer cells was mostly medium, but with large deviations; from very small (10%) to very high (up to 90%). However, there was a visibly lower ratio of immunoreactive cells compared with HPV− OPSCC. NHERF2 protein was localized exclusively in the cytoplasm in these cancer cells.

**Figure 4:**
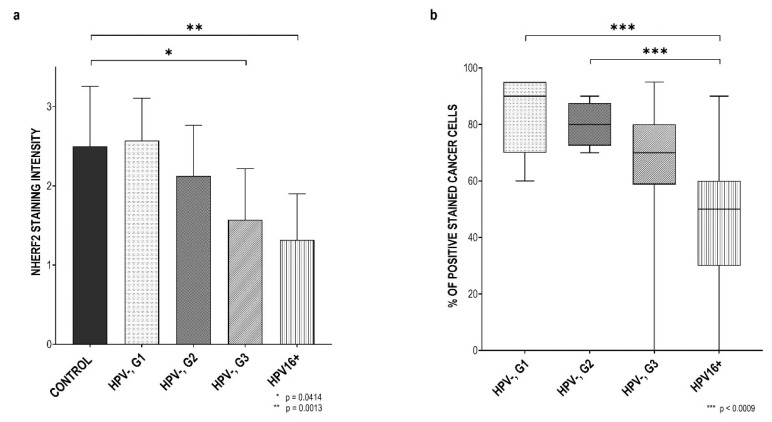
The intensity and ratio of NHERF2-positive staining in HPV− and HPV16+ OPSCCs. (**a**) The intensity of staining and (**b**) The proportion of NHERF2-positive cancer cells were observed by a pathologist and evaluated between HPV16+ and HPV− OPSCCs graded 1–3. Significance was determined using the Kruskal–Wallis statistical test and post hoc Dunn’s test, which were performed to reveal the difference in the level of staining intensity between groups with *p*-value (*) 0.0414, (**) 0.0013 and (***) < 0.0009.

Changes in the staining intensity and percentage of immunoreactive cancer cells were then compared between the HPV+ and HPV− tumors and the control group. As can be seen (Figure 4a), staining intensity decreased with the reduction of HPV− OPSCC differentiation. Although the sample size was rather small, the difference in staining intensity between the control group and HPV− G3 samples was statistically significant with a *p*-value of 0.0414. Greater variation was detected in the presence of HPV16, so this difference between the control tissue and HPV16+ OPSCCs was significant with a *p*-value of 0.0013, indicating that the presence of HPV16 E6 in these cancer types reduced the amount of NHERF2. In addition, the difference in the proportion of stained cancer cells in various cancer groups was examined (Figure 3b) and it can be seen that the ratio of immunoreactive cancer cells decreases in parallel with a reduction of differentiation i.e., in G2 and G3 HPV− OPSCCs, and this is even more visible in the presence of HPV16. In these, we noted a statistically significant difference between G1 HPV−OPSCCs and HPV16+ OPSCCs (*p* = 0.0005), as well as between G2 HPV− OPSCCs and HPV16+ OPSCCs (*p* = 0.0009). Although the difference between G3 HPV− OPSCCs and HPV16+ tumors was clearly visible, it was not statistically significant with this small number of tested samples. These results further confirm the observation that NHERF2 protein levels are lower in the presence of HPV16 in comparison with those in well and moderately differentiated HPV− OPSCCs, indicating the importance of NHERF2 regulation for HPV-mediated carcinogenesis in the head and neck.

## 4. Discussion

In the past 30 years, HPV infections have been characterized as a risk factor for the development of HNSCCs, especially OPSCCs, accounting for 50–80% of these tumors [48]. Interestingly, HPV-induced OPSCCs appear to be epidemiologically, clinically, anatomically, and even biologically, distinct from HPV− cancers at this anatomical site. Despite their increased prevalence, HPV-induced cancers have a favorable prognosis, possibly due to their outstanding treatment sensitivity [49] and the lower incidence of distant metastases [50]. However, the prediction of disease development, early detection of primary tumors, and prevention of malignant progression are still needed for survival rates to improve. Nowadays, approximately 4.5% of cancers worldwide are attributable to HPVs, with HPV16 being regularly the most frequently found type [51,52]. Although HPV infection is the most common sexually transmitted viral infection, in most cases the immune system rapidly resolves it without any long-term effects; but in cases of chronic infections, individuals are at an increased risk for cancer development. So far, the concept of HPV carcinogenesis includes deregulation of the E2 protein, either by disruption due to the HPV genome integrating into the host genome, or by methylation [21]. This is then followed by uncontrolled expression of the E6 and E7 oncoproteins which interfere with a plethora of cellular targets, primarily the two essential tumor suppressors p53 and pRb, which regulate normal cellular proliferation and homeostasis [20]. A unique characteristic of all HR E6 oncoproteins is their ability to target a number of cellular PDZ domain-containing proteins, including DLG1, DLG4, SCRIB, MAGI, PTPN13, and NHERF1. These interactions were shown to be important for the productive viral life cycle, while their loss is a characteristic of late stages in HPV-induced malignancies [27,29,31]. A recent study identified NHERF2 as a novel PDZ domain-containing target of HPV16 and HPV18 E6s, which induce its proteasomal degradation [37]. More interestingly, loss of NHERF2 appears to lead to p27 downregulation, which is a hallmark of many cancers [35,36]. Thus, we sought to investigate whether NHERF2 modulations can be associated with oncogenesis in the head and neck region, and to clarify the role of HPV in this process. In order to define these potential differences, we decided to monitor NHERF2 changes in HPV16+ and HPV− oropharyngeal tumors.

We did not observe any changes in NHERF2 localization in any of the groups tested, compared with the control group of uninfected tonsils. Interestingly, a previous study showed that NHERF1 with a mutation in the PDZ2 domain changes its localization in breast cancer [53]; since we have not observed a similar phenotype with NHERF2, this indicates that modifications in NHERF2 might have exclusive roles in carcinogenesis. Likewise, it should be considered that potential mutations in NHERF2 protein were not examined in the current study, so we can only speculate that these possible mutations may rest outside the PDZ2 domain and therefore do not necessarily promote changes in localization. Likewise, some post-transcriptional and post-translational modifications might have occurred, potentially affecting many aspects of protein function, including assembly, lifespan, interactions, molecular trafficking, solubility, and protein localization, which could explain the observed phenotype [54]. In addition, we noticed a gradual loss of the NHERF2 protein which correlated with a decrease in the differentiation of HPV− OPSCCs. Such results are not surprising, considering that they could indicate a greater number of accumulated mutations. This has been shown in a previous study to be the case with NHERF1, which is frequently mutated in breast cancers, while its complete loss was observed in estrogen receptor-negative cancers [55]. Interestingly, although G3 HPV− OPSCCs are morphologically similar to HPV16+ OPSCCs, with both cancer types exhibiting poorly differentiated to undifferentiated phenotypes, the decrease in NHERF2 expression was even more evident in the presence of HPV16. This observation closely follows the results of a study showing that HPV16 binds NHERF2 and directs it to proteasome-mediated degradation in cultured cells, thereby causing an overall reduction in NHERF2 protein levels [37]. Such protein degradation, caused by the presence of HPV16 E6, has also been observed for NHERF1 in cell lines derived from cervical carcinomas, and in HPV16+ cervical lesions [30]. Interestingly, we observed a statistically significant decrease in NHERF2 protein levels in the control group and in both G3 HPV− and HPV16+ OPSCC, suggesting that NHERF2 loss could play a role in increased proliferation that promotes tumorigenesis. In addition to a decrease in its protein level, we also observed a decrease in NHERF2 index or percentage of immunoreactive cancer cells with the higher grade of HPV− OPSCCs, but this was even more pronounced in the presence of HPV16. Although we did not observe an unequivocal result of the reduction in NHERF2 exclusively in the presence of HPV16, we can assume that even these small changes in expression could lead to an increase in cell proliferation that is associated with carcinogenesis, considering that NHERF2 serves as a negative regulator of cell proliferation [34,56]. Likewise, PDZ/PBM interactions are very specific, and a difference in just one amino acid within the PBM can greatly affect the affinity and strength of these interactions [57]. Furthermore, it has been shown that HPV16 E6 preferentially targets SCRIB [40,58] among PDZ domain-containing substrates, so it is likely that the remaining or unbound E6 oncoprotein would then interact with other cellular PDZ proteins, including NHERF2, with which it has a lower binding affinity. However, due to the small sample size, we cannot claim that the observed changes in NHERF2 expression patterns are sufficient to either distinguish OPSCCs by HPV status, or to predict disease progression. Overall, the results from this study indicate that certain variations in NHERF2 expression are likely to correlate with higher grades of OPSCCs, with these variations being more apparent in the presence of HPV16 E6. Nevertheless, further molecular assays and analyses on larger sample sizes are necessary to acquire a better understanding of NHERF2 molecular functions in the presence and absence of HPV. A greater cohort could be used to link changes in these functions to OPSCC onset, response to treatment, and overall survival.

## 5. Conclusions

To our knowledge, this is the first study investigating NHERF2 expression patterns in the process of oropharyngeal carcinogenesis. We provided new information about alterations in NHERF2 protein expression levels in HPV16+ and HPV− OPSCCs of three various grades. We observed a significant decrease in NHERF2 expression levels in poorly differentiated G3 HPV− and HPV16+ tumors. Interestingly, this effect of NHERF2 downregulation was markedly more evident in HPV16+ tumors. We also showed a significant decrease in NHERF2 immunoreactive cancer cells in HPV16+ samples suggesting its importance in HPV-driven oropharyngeal carcinogenesis.

## Data Availability

The data that support the findings of this study are available on reasonable request from the corresponding author. The data are not publicly available due to considerations of privacy.
